# Measuring what gets done: Using goal attainment scaling in a vocational counseling program for survivors of childhood cancer

**DOI:** 10.1002/cam4.5576

**Published:** 2023-02-12

**Authors:** Jason D. Pole, Barb Williams, Giancarlo Di Giuseppe, Sharon Guger, Elaine Stasiulis, Mark L. Greenberg, Brenda J. Spiegler, Kim Edelstein

**Affiliations:** ^1^ Centre for Health Services Research The University of Queensland Brisbane Queensland Australia; ^2^ Dalla Lana School of Public Health University of Toronto Toronto Ontario Canada; ^3^ Pediatric Oncology Group of Ontario Toronto Ontario Canada; ^4^ Department of Psychology Hospital for Sick Children Toronto Ontario Canada; ^5^ Research Institute, The Hospital for Sick Children Toronto Ontario Canada; ^6^ Institute of Medical Science, University of Toronto Toronto Ontario Canada; ^7^ Private Practice Toronto Ontario Canada; ^8^ Department of Supportive Care Princess Margaret Cancer Centre Toronto Ontario Canada; ^9^ Department of Psychiatry, Faculty of Medicine University of Toronto Toronto Ontario Canada

**Keywords:** goal attainment scaling, neoplasms, pediatrics, routinely collected health data, survivors, cancer

## Abstract

**Background:**

Childhood cancer survivors face education and employment challenges due to physical, cognitive, and psychosocial effects of the disease and treatments, with few established programs to assist them. The objectives of this study were to describe the implementation of Goal Attainment Scaling (GAS) to evaluate an educational and vocational counseling program established for survivors of childhood cancer, and analyze patterns of program engagement and client outcomes, stratified by demographic and diagnostic characteristics.

**Methods:**

A population‐based retrospective cohort study of childhood cancer survivors who were engaged with the Pediatric Oncology Group of Ontario's School and Work Transitions Program (SWTP) between January 2015 and December 2018 was utilized. Survivors were followed from SWTP engagement until May 30, 2019 to capture goal attainment. Individual goals were summarized across various demographic, disease, and treatment strata.

**Results:**

In total, 470 childhood cancer survivors (median age = 17.9, 58% male) set 4,208 goals in the SWTP during the study period. The mean length of observation was 130.8 weeks (SD = 56.9). Overall, 68% of the goals were achieved. Eighty‐three percent of the goals related to further education. Clients diagnosed with a solid tumor set the most goals on average, followed by those with central nervous system tumors and leukemia/lymphoma.

**Conclusions:**

The SWTP assists childhood cancer survivors in realizing their academic and vocational goals. Application of GAS in this setting is a feasible way to evaluate program outcomes. From the volume and breadth of the GAS goals set and achieved, the overall success of the SWTP appears strong.

## Graphical abstract

Childhood cancer survivors face education and employment challenges due to physical, cognitive, and psychosocial effects of the disease and treatments, with few established programs to assist them. The School and Work Transitions Program assists childhood cancer survivors in realizing their academic and vocational goals. Application of Goal Attainment Scaling in this setting is a feasible way to evaluate program outcomes.

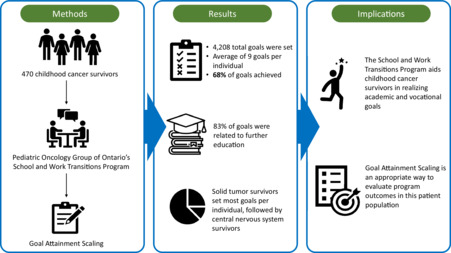

## 1 BACKGROUND

Most children diagnosed with cancer in developed nations will be cured.^1^ Consequently, the size of the childhood cancer survivor population continues to expand. It is estimated that there are over 400,000 childhood cancer survivors (0.11% of the population) in the United States and approaching 500,000 in Europe, of whom 24% have survived more than 30 years.^2^, ^3^, ^4^ In Ontario, Canada, the childhood cancer survivor population has been estimated to be 19,900 as of 2017.^5^ Childhood cancer survivors are at an elevated risk for the development of multiple and diverse chronic health conditions.^6^, ^7^

Childhood cancer survivors are also at increased risk for difficulty achieving success in employment and education, related to neurocognitive changes as a result of the disease or its treatment.^8^, ^9^ Neurocognitive problems affecting learning and memory may be exacerbated by physical, emotional, and psychosocial effects of individual cancer experiences.^10^, ^11^, ^12^

The Pediatric Oncology Group of Ontario (POGO), a collaborative network and official advisor to the provincial health system on childhood cancer control, launched what is currently called the School and Work Transitions Program (SWTP) in 2002,^13^ to provide support for childhood cancer survivors in Ontario, Canada. The SWTP provides survivors of childhood cancer (hereafter referred to as “clients”) support to mitigate barriers to education and vocational success. Services are provided largely through individual counseling but also through presentations, publications, and workshops for clients and other professionals; development of positive community partnerships; advocacy work and ongoing research to ensure continuous improvement of service to its childhood cancer survivor population.^14^, ^15^

Individual client needs and the diverse goals and client specific outcomes require tailoring of support services and render the measurement of SWTP success difficult. Although the SWTP demonstrated qualitative evidence of success in a small group of clients,^16^ as the program grew it became more challenging to capture differences in service provision across counselors and sites, and consistent monitoring and evaluation of the program's success was lacking.

In an effort to better track individualized goals and related outcomes of each client, identify similarities and differences across clients, and to act as markers of success, the SWTP implemented Goal Attainment Scaling (GAS^17^) in 2013. GAS, first introduced in the late 1960s, was developed as a general method to evaluate mental health outcomes. Soon, the general applicability of GAS as a measurement and evaluation tool for a variety of healthcare service delivery outcomes was realized. At its core, GAS enables quantitative analysis of qualitative data (i.e., measures personally meaningful change) resulting from many forms of intervention. GAS requires outcome scales to be developed that are tailored to the individual and specific to the context in which it is being applied. GAS has been extensively utilized in various aspects of rehabilitation across various age and patient groups including adult brain tumor patients.^18^, ^19^, ^20^, ^21^, ^22^

The objectives of this study were to describe the implementation of GAS in the SWTP, analyze patterns of program engagement, and use GAS to measure outcomes among SWTP clients, stratified by demographic and diagnostic characteristics.

## 2 METHODS

In this population‐based retrospective cohort study, participants were childhood cancer survivors who were referred to and initially engaged (defined as responding to an initial contact request by a SWTP counselor) with the SWTP between January 1, 2015 and December 31, 2018. Participants were followed from date of initial engagement until May 30, 2019. This project was approved by the Research Ethics committee at the University of Toronto (Protocol No. 37234). This study is a secondary analysis of routinely collected data and was completed under a waiver of consent.

Referrals to the SWTP come through Pediatric AfterCare Clinics at tertiary hospitals throughout Ontario (specially designed medical clinics for childhood cancer survivors), other health care providers, and self‐referral. SWTP counselors regularly attend AfterCare Clinics to ensure clinic staff and potential clients have an understanding of the program and services offered to encourage and facilitate client referrals. The referral process requests that one or more referral reasons, selected from five broad areas (secondary school, post‐secondary school, employment/volunteerism, scholarships, or community services) be identified which are then prioritized by the counselor and client. Following referral, potential clients are contacted by one of the SWTP counselors in the province and enrolled in the program if they are interested. Counselors work with clients to define and support their academic, vocational, or community services support goal(s) via in‐person meetings, telephone calls, or through electronic correspondence. Clients with SWTP experience prior to 2015 were excluded from analyses as the GAS system was not fully implemented. Referred clients who chose not to engage with the program after initial contact were also excluded since no goals were set. The timing and amount of support provided to clients varies as client needs change.

GAS was used to quantify clients' progress in achieving goals. SWTP counselors worked with clients to set feasible goals that were client actions and were agreed upon by both client and counselor. Generally, the goals were personally meaningful and followed the SMART framework, that is, specific, measurable, attainable, relevant, and time‐bound.^23^ In this way, clients were aware of what was expected of them but were not explicitly informed that their actions were being scored, this ensured that clients did not feel self‐conscious about being monitored or scored and given we were using GAS to evaluate program outputs and not individual clients. A default time frame of 2 weeks for goal attainment was the norm, with adjustment for circumstances that might demand a different time frame (e.g., college application deadlines). There were no restrictions on the number of goals a counselor could set with an individual client. Example goals include preparing/drafting a resume over 2 weeks or developing and submitting applications for post‐secondary education over 4 weeks. Three levels of attainment from 0 (target reached) to +2 (achievement beyond the specified goal), and two levels of insufficiency −1 (some achievement but goal not reached) to −2 (no action toward goal) were specified. If the time elapsed without the client completing the goal, the counselor scored the goal −2 and would either re‐establish the same goal with the client at the next interaction or reassess the client's ability to meet the original goal and modify it by breaking the goal into smaller actions to assist the client with attainment.

Members of the implementation team and all counselors underwent a one‐day training session on the development and implementation of GAS goals. During this face‐to‐face, hands‐on training counselors were able to develop goals using client scenarios, followed by group discussion on how the goals could be improved. As part of the SWTP, counselors meet regularly to discuss program issues and during the preliminary implementation phase, time during these meetings was devoted to collectively discussing GAS goals. Any questions that were not able to be addressed among their own community of practice were discussed with the implementation team. As new counselors joined the program, the SWTP Manager provided in‐depth orientation and training to GAS goal setting to ensure consistency across the program.

To begin using GAS to measure SWTP outcomes, all client goals set in a preliminary period of 6–8 months were examined by two members of the implementation team (JDP and BW) to identify common domains of practice and goals set with clients across sites. The creation of standard goals was necessitated by counselor time constraints and the associated additional time needed to create each GAS goal de novo. After reviewing several hundred GAS goals set by five counselors, repetition in goals was evident. From this effort, 15 standard goals were established (Table 1). The inclusion of the last standard goal permits the counselor to create a new goal should they determine that none of the standard goals are appropriate. In addition, four broad practice domains were identified to assist in collecting information on the context of the individual goals; (1) community resource, (2) education, (3) employment/volunteering, and (4) SWTP engagement. Each domain contained sub‐domains, such as academic accommodation or course completion within the education domain, full details are provided in Table 4. These domains, sub‐domains, and goals were integrated into the SWTP electronic charting system, so that counselors selected each via a series of drop‐down menus and set the date for goal adjudication. This permitted the charting system to send electronic reminders to counselors to score the goal at the appropriate intervals.

**TABLE 1:** Standardized GAS goals and scores for the SWTP program.

	Score				
Standard Goal	−2	−1	0	+1	+2
1 Client replies by email/phone to SWTP outreach	Client does not respond to SWTP outreach	Client parent/guardian responds to SWTP outreach	Client responds to SWTP outreach	Client responds to SWTP and sets direction for next steps	Client responds to SWTP and has some information/research to share with SWTP
2 Client responds to SWTP Counselor e‐mail	Client does not respond to e‐mail	Client does not address the relevant content of e‐mail	Client responds appropriately to SWTP counselor e‐mail	Client responds and generates further discussion/asks more questions	Client responds to e‐mail and sets future meeting
3 Client meets with SWTP counselor (phone or in‐person)	Client does not meet with SWTP counselor	Client contacts SWTP to re‐schedule meeting	Client meets with SWTP counselor	Client meets with SWTP counselor and has prepared questions	Client meets with SWTP counselor and has extensive questions and/or relevant documentation
4 Client meets with SWTP and brings requested documents	Client does not attend meeting	Client brings some but not all requested documents to SWTP meeting	Client attends meeting with all requested documents	Client attends meeting with all requested documents plus additional information	Client attends meeting with all requested documents, additional information and prepared questions
5 Client attends meeting /makes contact with external agency/organization/institution	Client does not contact/meet with organization.	Client attempted contact but did not actually make contact or did not procure required information from person/office	Client attends meeting/makes contact with external agency/organization/institution	Client attends/makes contact and advises SWTP of status	Client attends/makes contact with extensive questions and has collected relevant documentation
6 Client makes post‐secondary program selection(s)	Client has not made any selection(s)	Client has narrowed down choices by not committed to program selection(s)	Client has made post‐secondary program selection(s)	Client has made selections and contacts SAVIT Counselor for application assistance.	Client has applied to selected program(s)
7 Client submits application.	Client does not submit application	Client calls SWTP with questions in order to submit application	Client submits application.	Client submits application and contacts counselor to advise of status	Client submits application and has researched additional opportunities or completed interview
8 Client completes application forms	Client does not complete application form	Client calls SWTP with questions in order to complete application	Client completes application.	Client completes application and contacts counselor to advise of status	Client completes and submits application
9 Client makes contact with SWTP Counselor to discuss post‐secondary education material provided	client did not contact SWTP Counselor	Client makes contact but has not reviewed material/done homework	Client has reviewed material provided /done homework	reviewed material/done homework and came prepared with questions	Client has reviewed provided material/done homework and has made decisions or did personal extensive research
10 Client enrolls in required prerequisite courses	Client has not enrolled for course(s)	Client has investigated course selection with guidance counselor	Client enrolls in identified required prerequisite courses	Client enrolls in courses and has explored additional complimentary work/school course opportunities	Client enrolls in courses and has registered/applied for additional complimentary work/school course opportunities
11 Client completes self‐assessment tool	Client does not complete self‐assessment tool.	Client calls SWTP with questions in order to complete tool	Client completes self‐assessment tool	Client completes tool and contacts counselor to discuss	Client completes tool and has researched results/provided further exploration results
12 Client completes draft/corrections to essay/cover letter/resume	Client does not complete draft or corrections	Client completes some parts or some corrections	Client completes draft/corrections to essay/cover letter/resume	Client completes and provides up‐dated version to SWTP counselor	Client completes and has friends/additional people review/has applied for job
13 Client completes research for available jobs	Client does not complete research	Client calls SWTP with questions in order to complete search	Client completes research for available jobs	Client has completed search and started preparing job application	Client has completed search and applied for position
14 Client completes course requirements	Client does not complete course requirements	client contacts SWTP about issues/challenges around completing course requirements	Client completes course requirements	Client completes course requirements and advises SWTP of status	Client completes course requirements and has explored next steps
15 Create your own goal					

### 2.1 Data sources

There were two main sources of information for this analysis. First, the SWTP collected detailed records via a custom electronic charting system that assists in client record management to capture details of the GAS goals set and assessed. Cancer diagnosis and treatment information for SWTP clients was obtained from the POGO Networked Information System (POGONIS).

### 2.2 Analysis

Overall, the analysis as planned was descriptive in nature with the number, type, and outcome of the GAS goals examined and stratified by demographic and disease factors. The analysis stratified participants into three broad diagnostic groups according to the first primary cancer (leukemia/Lymphoma, central nervous system (CNS) tumors, and other extracranial solid tumors). These groups were selected due to general physical and cognitive deficits experienced as resulting from diagnosis and treatment. Length of engagement with SWTP was examined by categorizing the duration into five periods: 0–<1, 1–<2, 2–<3, 3–<4, and 4+ years. Given that the length of engagement with SWTP was thought to impact the number and type of goals set, a stratified analysis was undertaken that examined the outcomes in the first and second half of the total length of SWTP engagement. Only clients that had GAS goals set within both first‐half and second‐half of their engagement period were considered for the stratified split‐half analysis. Raw GAS scores were converted to T‐scores following the Keresuk and Sherman method before all analyses.^24^ Briefly, T‐scores were calculated assuming an equal weight for each goal and accounting for the number of goals included in each T‐score estimate (which varies based on the number of goals by each client or within various strata). Therefore, the T‐score is a linear transformation of the sum of the individual goal scores represented on a scale from 0 to 100, with a mean of 50 and standard deviation of 10.

## 3 RESULTS

There were 470 study participants (clients) who had their first referral and engaged with SWTP between January 1, 2015 and December 31, 2018. Of these, 259 participants had multiple engagements (re‐engagement) with SWTP over the period of observation.

Table 2 provides demographic and disease characteristics of the study participants stratified by diagnostic group. The majority of participants were diagnosed with leukemia/lymphoma (56.0%) followed by CNS (22.8%) and extracranial solid tumors (21.2%). At the time of SWTP referral, the median age was 17.9 years and this was consistent across diagnostic categories. Overall, the median age at diagnosis was 7.7 years, but this ranged from 6.1 years for the non‐CNS tumors to 10.9 years for the CNS tumors. Relapsed disease occurred in 11.5%, with slightly higher relapse proportions noted in the CNS and solid tumor groups. Of the participants who did not have CNS tumors, 56.2% received CNS‐directed therapy, defined as intrathecal chemotherapy and/or cranial radiotherapy. The proportion of CNS‐directed therapy was highest in participants diagnosed with leukemia/lymphoma (71.5% overall; 90.7% among acute lymphoblastic leukemia patients and 78.6% among non‐Hodgkin lymphoma patients), than solid tumors (16.0%). Just over 11% of participants received a hematopoietic stem cell transplant (HSCT). Forty‐seven percent of the HSCTs were allogeneic and all occurred among patients in the leukemia/lymphoma group.

**TABLE 2:** Population characteristics and outcomes of *n* = 470 SWTP clients who engaged in the program with first referral date between 01JAN2015 and 31DEC2018.

Characteristic	Diagnostic group of primary cancer							
	Total		Leukemia/Lymphoma		CNS		Solid tumor + other	
	*N*	(%)	*N*	(%)	*N*	(%)	*N*	(%)
Total cases	470	100	263	100.0	107	100	100	100
Sex								
Male	273	58.1	164	62.4	62	57.9	47	47.0
Female	197	41.9	99	37.6	45	42.1	53	53.0
Age at SWTP Interview								
yrs (median, IQR)	17.9	(17.2–19.3)	17.9	(17.3–18.9)	17.6	(17–19.3)	18.0	(17.4–19.9)
< 19 yrs	342	72.8	202	76.8	75	70.1	65	65.0
19–22 yrs	68	14.5	29	11.0	18	16.8	21	21.0
23+ yrs	60	12.8	32	12.2	14	13.1	14	14.0
Time since diagnosis								
yrs (median, IQR)	11.6	(5.6–15.2)	12.0	(6.3–15.1)	9.2	(5.3–13)	13.2	(5.4–16.5)
< 5 yrs	101	21.5	56	21.3	24	22.4	21	21.0
5–9 yrs	94	20.0	41	15.6	35	32.7	18	18.0
10–14 yrs	151	32.1	99	37.6	32	29.9	20	20.0
15+ yrs	124	26.4	67	25.5	16	15.0	41	41.0
Age at diagnosis								
yrs (median, IQR)	7.7	(3.7–12.8)	6.2	(3.8–13.3)	10.9	(5.9–12.9)	6.1	(2.1–12.5)
< 1 yrs	21	4.5	6	2.3	1	0.9	14	14.0
1–4 yrs	144	30.6	94	35.7	21	19.6	29	29.0
5–9 yrs	117	24.9	70	26.6	28	26.2	19	19.0
10–14 yrs	118	25.1	51	19.4	40	37.4	27	27.0
15+ yrs	70	14.9	42	16.0	17	15.9	11	11.0
Diagnosis year								
< 1990	8	1.7	5	1.9	1	0.9	2	2.0
1990–1999	61	13.0	29	11.0	9	8.4	23	23.0
2000–2009	259	55.1	157	59.7	59	55.1	43	43.0
2010+	142	30.2	72	27.4	38	35.5	32	32.0
Relapsed disease								
Yes	54	11.5	27	10.3	14	13.1	13	13.0
No	416	88.5	236	89.7	93	86.9	87	87.0
Secondary malignancy diagnosis								
Yes	12	2.6	3	1.1	5	4.7	4	4.0
No	458	97.5	260	98.9	102	95.3	96	96.0
Treatment modality (for any cancer)								
Any treatment	453	96.4	253	96.2	100	93.5	100	100.0
Chemotherapy	407	86.6	253	96.2	63	58.9	91	91.0
Intrathecal chemo	184	39.2	182	69.2	1	0.9	1	1.0
Antimetabolites	225	47.9	206	78.3	6	5.6	13	13.0
Anthracyclines	266	56.6	207	78.7	1	0.9	58	58.0
Alkylating agents	264	56.2	180	68.4	32	29.9	52	52.0
Epipodophyllotoxins	128	27.2	67	25.5	10	9.4	51	51.0
Platinum agents	86	18.3	4	1.5	41	38.3	41	41.0
Radiation	165	35.1	69	26.2	46	43.0	50	50.0
Cranial	106	22.6	44	16.7	46	43.0	16	16.0
Non‐cranial	59	12.6	25	9.5			34	34.0
Surgery	195	41.5	44	16.7	71	66.4	80	80.0
Hematopoietic stem cell transplant	53	11.3	31	11.8	12	11.2	10	10.0
Autologous	28	6.0	6	2.3	12	11.2	10	10.0
Allogeneic	25	5.3	25	9.5				
CNS‐directed therapy (IT chemo or cranial rads)	251	53.4	188	71.5			16	16.0

When considering differences in demographic and disease characteristics by length of engagement with SWTP (data not shown), those participants with engagements of 4+ compared to 1–2 years had a higher proportion of males (63.8% vs. 53.7%) and a diagnosis of leukemia/lymphoma (61.7% vs. 50.4%). There was a lower proportion of solid tumors (19.2% vs. 25.6%) and the median age at diagnosis was also lower (5.5 years vs. 9.8 years) in the 4+ versus 1–2 years engagement groups.

Table 3 outlines the detailed GAS outcomes overall and stratified by domain and diagnostic group. In total, 4,208 goals were set among the 470 participants. On average, participants were followed for 130.8 weeks (SD = 56.9) setting an average of 9.0 goals per participant with 68% of these goals being achieved or surpassed (defined as a GAS score ≥0 or a T‐score ≥ 50). Educational goals were set by most participants (83%), followed by SWTP engagement (55%) (reflecting re‐engagement), employment/volunteering (29%), and community resources (8%).

**TABLE 3:** GAS outcomes of *n* = 470 SWTP clients who engaged in the program with first referral date between 01JAN2015 and 31DEC2018.

Characteristic	Diagnostic group of primary cancer											
	Total			Leukemia/Lymphoma			CNS			Solid tumor + other		
	μ	SD	Range	μ	SD	Range	μ	SD	Range	μ	SD	Range
Overall												
Clients setting goals, *n*	470			263			107			100		
Amount of goals set, *n*	4208			2047			1010			1151		
Achieved GAS goal (*n*, %)*	2861	(68%)		1375	(67%)		678	(67%)		808	(70%)	
Mean No. of goals set	9.0	(12.1)	(1–139)	7.8	(11.2)	(1–139)	9.4	(9.7)	(1–48)	11.5	(15.9)	(1–98)
GAS score	42.5	(10.4)	(19.9–74.6)	42.3	(10.6)	(19.9–74.6)	43.3	(10.9)	(19.9–73.5)	42.3	(9.3)	(22.6–62.9)
Domain												
Community resources												
Clients setting goals (*n*, %)	39	(8%)		13	(5%)		17	(16%)		9	(9%)	
Amount of goals set, *n*	84			20			46			18		
Achieved GAS goal (*n*, %)*	52	(62%)		12	(60%)		29	(63%)		11	(61%)	
Mean No. of goals set	2.2	(1.7)	(1–7)	1.5	(0.9)	(1 ‐ 3)	2.7	(1.9)	(1–7)	2.0	(1.9)	(1–7)
GAS score	41.1	(10.6)	(25.2–60)	44.9	(10.1)	(30–60)	39.1	(11.3)	(25.2–56.2)	39.4	(9.4)	(30–50)
Education												
Clients setting goals (*n*, %)	388	(83%)		215	(82%)		93	(87%)		80	(80%)	
Amount of goals set, *n*	2837			1411			711			715		
Achieved GAS goal (*n*, %)*	1964	(69%)		986	(70%)		479	(67%)		499	(70%)	
Mean No. of goals set	7.3	(10.2)	(1–127)	6.6	(10.7)	(1–127)	7.7	(7.9)	(1–44)	8.9	(11.0)	(1–59)
GAS score	43.3	(10.8)	(21.6–76.3)	43.5	(10.7)	(21.6–76.3)	43.5	(11.7)	(22.6–73.5)	42.8	(10.0)	(22.6–65.8)
Employment/Volunteer												
Clients setting goals (*n*, %)	137	(29%)		76	(29%)		26	(24%)		35	(35%)	
Amount of goals set, n	675			314			110			251		
Achieved GAS goal (n, %)*	444	(66%)		194	(62%)		79	(72%)		171	(68%)	
Mean No. of goals set	4.9	(6.6)	(1–57)	4.1	(4.8)	(1 ‐ 26)	4.2	(3.6)	(1 ‐ 13)	7.2	(10.3)	(1–57)
GAS score	42.4	(10.3)	(25.2–70)	41.5	(11.3)	(25.2–70)	45.0	(8.2)	(30–60)	42.4	(9.1)	(29.3–60.2)
SWTP engagement												
Clients setting goals (*n*, %)	259	(55%)		135	(51%)		66	(62%)		58	(58%)	
Amount of goals set, *n*	612			302			143			167		
Achieved GAS goal (*n*, %)*	401	(66%)		183	(61%)		91	(64%)		127	(76%)	
Mean No. of goals set	2.4	(2.0)	(1 ‐ 13)	2.2	(1.7)	(1 ‐ 10)	2.2	(2.0)	(1 ‐ 10)	2.9	(2.5)	(1 ‐ 13)
GAS score	43.8	(9.6)	(22.6–70)	43.0	(9.7)	(22.6–70)	43.8	(9.4)	(22.6–60)	45.6	(9.5)	(25.2–61.6)
Split half analysis**, *n*	394			216			95			83		
Early												
Amount of goals set, n	2356			1083			563			710		
Achieved GAS goal (*n*, %)*	1658	(70%)		742	(69%)		390	(69%)		526	(74%)	
Mean No. of goals set	6.0	(7.9)	(1–79)	5.0	(7.0)	(1–79)	5.9	(6.4)	(1 ‐ 31)	8.6	(10.9)	(1–64)
GAS score	43.8	(10.3)	(22.6–75.4)	43.1	(10.3)	(22.6–68.1)	44.5	(10.7)	(23.9–75.4)	44.6	(10.0)	(22.6–70)
Weeks of engagement	12.1	(12.2)	(0–53)	11.5	(12.4)	(0–52)	13.3	(11.9)	(0–53)	12.2	(11.8)	(0–51)
Late												
Amount of goals set, *n*	1772			915			433			424		
Achieved GAS Goal (*n*, %)*	1156	(65%)		604	(66%)		278	(64%)		274	(65%)	
Mean No. of goals set	4.5	(5.9)	(1–60)	4.2	(5.9)	(1–60)	4.6	(5.0)	(1 ‐ 23)	5.1	(6.6)	(1 ‐ 34)
GAS Score	42.0	(11.3)	(22.6–76.1)	42.0	(11.3)	(22.6–76.1)	42.7	(12.2)	(22.6–70)	41.4	(10.1)	(22.6–62.4)
Weeks of engagement	12.3	(12.2)	(0–54)	11.7	(12.4)	(0–53)	13.5	(12.0)	(0–54)	12.4	(11.8)	(0–52)

* Achieved goal defined as a GAS score ≥ 0 versus <0.

** Split half analysis outcomes using *n* = 394 clients who utilized SWTP long enough to permit split‐half analysis.

The solid tumor group set the highest average number of goals (11.5 goals per person) followed by the CNS group (9.4) then the leukemia/lymphoma group (7.8). Goal achievement was similar among the diagnostic groups ranging from 67–70% achievement. The CNS group set the highest proportion of community resources goals (16%) followed by solid tumors (9%), and leukemia/lymphoma (5%), with each group having similar levels of goal achievement. Similarly, the CNS group set the highest proportion of education goals (87%) followed by leukemia/lymphoma (82%) and solid tumors (80%), although the CNS group achieved less of their educational goals (67% of goals achieved) compared to the other groups (each with 70%). The solid tumor group set the highest proportion of employment/volunteer goals (35%), followed by leukemia/lymphoma (29%) and solid tumors (24%) with the leukemia/lymphoma group having lowest proportion of goal achievement (62%) followed by solid tumors (68%) and CNS (72%).

When examining GAS outcomes stratified by age at initial engagement with SWTP (data not shown), there was little difference in achievement by age (68% for those <19; 64% for 19–22; 72% for 23+ years old). The 19–22 years old set the highest proportion of community resources goals (3.3%) followed by those aged 23+ (3.1%) and then the <19 (1.6%). Those aged <19 year set the highest proportion of education goals (74.5%) followed by 19–22 (59.7%) and then the 23+ years old (39.5%). Those 23+ years old set the highest proportion of employment/volunteer goals (45.3%) followed by those aged 19–22 years (21.6%) and then the <19 years old (9.4%).

The split‐half analysis assessing if the length of engagement with SWTP impacted on the number and type of goals set, utilized only 394 participants (some participants did not set goals in the first and second half of their engagement with SWTP therefore not permitting analysis). Overall, participants were setting 1.3 times more goals in the earlier period compared to the later with the greatest difference in the solid tumor group (1.7 times), followed by the CNS group (1.3 times), and the leukemia/lymphoma group (1.2 times). The proportion of achieved goals is similar comparing the early to later period in the leukemia/lymphoma group (69% vs. 66%), with small differences in the CNS group (69% vs. 64%), and the largest difference in the solid tumor group (74% vs. 65%).

Table 4 presents a similar analysis of the GAS outcomes, but examines each sub‐domain stratified by diagnosis group. Overall, when considering goals set in the education domain, 29% (811/2837) were for post‐secondary exploration and 20% were for scholarship applications. When examining by diagnostic group, the proportion of post‐secondary exploration goals was greater than scholarship application for the leukemia/lymphoma and CNS groups (32% and 19% and 28% and 16%, respectively) but was reversed for the solid tumor group (22% and 27%). Overall, when considering goals set in the employment/volunteer domain, 30% (200/675) were for employment support. When examining by diagnostic group, the proportion of employment support goals was similar for the leukemia/lymphoma (31%) and solid tumor group (32%) but lower for the CNS group (22%).

**TABLE 4:** GAS outcomes for each sub‐domain of *n* = 470 SWTP clients who engaged in the program with first referral date between 01JAN2015 and 31DEC2018.

Characteristic	Diagnostic Group of primary cancer															
	Total				Leukemia/Lymphoma				CNS				Solid tumor + other			
	No. clients	No. goals	Avg. goals	Met GAS Goal	No. clients	No. goals	Avg. goals	Met GAS Goal	No. clients	No. goals	Avg. goals	Met GAS Goal	No. clients	No. goals	Avg. goals	Met GAS Goal
SWTP clients overall	470	4208	9.0	2861 (68%)	263	2047	7.8	1375 (67.2%)	107	1010	9.4	678 (67.1%)	100	1151	11.5	808 (70.2%)
Domain																
Community resources																
ODSP	9	21	2.3	12 (57.1%)	3	7	2.3	4 (57.1%)	5	13	2.6	8 (61.5%)	1	1	1.0	
Day program																
Social/recreational	17	21	1.2	12 (57.1%)	4	4	1.0	2 (50%)	8	11	1.4	6 (54.5%)	5	6	1.2	4 (66.7%)
Other community resources	18	42	2.3	28 (66.7%)	7	9	1.3	6 (66.7%)	7	22	3.1	15 (68.2%)	4	11	2.8	7 (63.6%)
Education																
Academic accommodation	100	330	3.3	226 (68.5%)	50	160	3.2	111 (69.4%)	32	97	3.0	67 (69.1%)	18	73	4.1	48 (65.8%)
Course application	28	52	1.9	37 (71.2%)	20	33	1.7	23 (69.7%)	4	10	2.5	5 (50%)	4	9	2.3	9 (100%)
Course completion	26	64	2.5	39 (60.9%)	10	21	2.1	14 (66.7%)	9	18	2.0	7 (38.9%)	7	25	3.6	18 (72%)
Post‐secondary exploration	204	811	4.0	552 (68.1%)	117	457	3.9	322 (70.5%)	48	200	4.2	131 (65.5%)	39	154	3.9	99 (64.3%)
Program application: College	67	207	3.1	163 (78.7%)	32	99	3.1	80 (80.8%)	17	50	2.9	35 (70%)	18	58	3.2	48 (82.8%)
Program application: University	55	128	2.3	95 (74.2%)	29	69	2.4	49 (71%)	11	29	2.6	23 (79.3%)	15	30	2.0	23 (76.7%)
Residence/housing application	11	23	2.1	18 (78.3%)	6	10	1.7	9 (90%)	4	9	2.3	6 (66.7%)	1	4	4.0	3 (75%)
Scholarship application	172	580	3.4	408 (70.3%)	81	272	3.4	189 (69.5%)	45	113	2.5	76 (67.3%)	46	195	4.2	143 (73.3%)
Student Loan application (eg. OSAP)	42	105	2.5	67 (63.8%)	22	67	3.0	47 (70.1%)	8	11	1.4	6 (54.5%)	12	27	2.3	14 (51.9%)
Other	112	238	2.1	161 (67.6%)	53	91	1.7	59 (64.8%)	33	74	2.2	51 (68.9%)	26	73	2.8	51 (69.9%)
Secondary school support	47	125	2.7	79 (63.2%)	22	61	2.8	33 (54.1%)	16	46	2.9	35 (76.1%)	9	18	2.0	11 (61.1%)
Post‐secondary school support	69	174	2.5	119 (68.4%)	28	71	2.5	50 (70.4%)	25	54	2.2	37 (68.5%)	16	49	3.1	32 (65.3%)
Employment/Volunteer																
Career exploration	25	49	2.0	34 (69.4%)	13	28	2.2	19 (67.9%)	5	10	2.0	7 (70%)	7	11	1.6	8 (72.7%)
Employment support	64	200	3.1	137 (68.5%)	32	96	3.0	56 (58.3%)	12	24	2.0	20 (83.3%)	20	80	4.0	61 (76.3%)
Position application	38	105	2.8	86 (81.9%)	20	52	2.6	41 (78.8%)	7	14	2.0	11 (78.6%)	11	39	3.5	34 (87.2%)
Position search	58	141	2.4	79 (56%)	30	73	2.4	37 (50.7%)	10	17	1.7	13 (76.5%)	18	51	2.8	29 (56.9%)
Resume preparation	34	133	3.9	77 (57.9%)	16	52	3.3	32 (61.5%)	9	33	3.7	18 (54.5%)	9	48	5.3	27 (56.3%)
Other employment/volunteer	30	47	1.6	31 (66%)	11	13	1.2	9 (69.2%)	7	12	1.7	10 (83.3%)	12	22	1.8	12 (54.5%)
SWTP Engagement																
Initial assessment of needs	259	612	2.4	401 (65.5%)	135	302	2.2	183 (60.6%)	66	143	2.2	91 (63.6%)	58	167	2.9	127 (76%)

## 4 DISCUSSION

The POGO's SWTP is a unique counseling support program for survivors of childhood cancer. Given that the structure of the program is fundamentally an individual one‐to‐one counseling experience, assessing global program outcomes is difficult. GAS was successfully introduced to the counseling service. SWTP created a standardized set of goals and integrated this into the electronic charting system, allowing us to characterize the academic and vocational goals of childhood cancer survivors, understand SWTP counselors' impact in helping survivors achieve their goals, and identify gaps in service provision. Application of GAS in this setting is feasible as evidenced by the high average number of goals set for each client.

The characteristics of the clients who engage with SWTP are reasonably representative of the childhood cancer survivor population in Ontario with the notable difference being the higher proportion of male clients. Whether this is due to referral bias, or that males seek the support offered by the SWTP more often than females is unclear and requires further research. Female participation has been more common in late‐effects research and counseling intervention programs for childhood cancer survivors.^25^, ^26^, ^27^, ^28^

When considering the pattern of goals set and the success of achieving them, it was surprising that the CNS group did not have the highest number of goals set or the lowest success. Given the often complex physical, cognitive, and social deficits that are associated with surviving a CNS tumor,^29^ and anecdotal evidence from the counselors, we would have expected a different pattern. However, the limited goals may reflect clients' lower achievement expectations and the successes attained may be due to the counselor's ability to break goals into smaller parts to facilitate achievement. It is encouraging to know that both the SWTP and the GAS system are flexible in implementation to be sensitive to the unique needs of this patient population. Similarly, as expected the leukemia/lymphoma patients set the smallest number of goals but unexpectedly had goal success similar to CNS patients and below solid tumor patients. Given many leukemia/lymphoma patients experience fewer relapses and secondary malignancies when compared to the solid tumor patients, we would have expected higher goal achievement in this group. Still, higher levels of exposure to CNS directed therapy compared to the solid tumor patients may impact goal achievement. Future research should explore possible mechanisms.

As expected, the number of goals set in the first half of the client engagement period was higher than the second half possibly owing to counselors making the initial goals smaller and less complex until they learn more about the individual client and the client's personal commitment to achieving the goals.

Previous work has identified that childhood cancers survivors utilize special education services nearly three times more frequently than sibling controls and overall educational attainment is reduced and related to specific diagnostic and treatment sub‐groups.^30^, ^31^, ^32^, ^33^, ^34^, ^35^, ^36^ It is clear when looking at the sub‐domains, client's access SWTP most often for assistance with post‐secondary education (e.g., exploring options, courses, and scholarship applications). Providing connection to post‐secondary education was one of the driving forces at the inception of the program. It appears that the SWTP is meeting this need.

The small number of goals set in the community resources domain should not be interpreted as a lack of interest by the clients for assistance in these areas. Although only a small number of clients set goals in this area, access to these community services is vital and can be complex. Clients may not be aware of available services and resources in the community and the SWTP program serves a special niche in providing clients with assistance in identifying and engaging with these difficult to access resources.

A 2015 review highlighted the special psychosocial, health‐promotion, and neurocognitive needs of adult survivors of childhood cancer.^12^ Within this systematic review the authors were not able to identify any studies that aimed at supporting educational or employment outcomes congruent with the goals of the SWTP. Identified studies examined neurocognitive interventions aimed at improving educational performance that most often focused on increasing working memory, cognitive flexibility, and attention as well as psychosocial interventions focusing on social competence. All interventions were short, lasting only hours to weeks and none provided the long‐term one‐to‐one counseling that is characteristic of the SWTP. A recent review examining education support noted substantial proportions of childhood cancer survivors received education support, but most supports lacked any evaluation of their effectiveness thereby calling into question their utility.^37^ Given the paucity of literature in this area, it is difficult to find outcome evaluations suitable for comparison to those presented herein.

The SWTP offered to survivors of childhood cancer in Ontario, Canada appears to be unique. The program has good engagement with clients, who come to the program with multiple goals. The overall outcome of the program appears positive, is valid across diagnoses and for several groups of issues. The success is likely a result of the one‐to‐one counseling model where client‐counselor relationships can drive individual progress toward education and employment goals to bolster and support survivors toward a productive and rewarding future. Similar programs to the SWTP delivering direct‐to‐client services to survivors of childhood cancer should be considered as part of routine long‐term follow‐up care.

## Data Availability

Research data are not shared.

## References

[1] Craft AW . Childhood cancer‐mainly curable so where next? Acta Paediatr. 2000;89(4):386‐392. 10830445 10.1080/080352500750028041

[2] Mariotto AB , Rowland JH , Yabroff KR , et al. Long‐term survivors of childhood cancers in the United States. Cancer Epidemiol Biomarkers Prev. 2009;18(4):1033‐1040. 19336557 10.1158/1055-9965.EPI-08-0988

[3] Phillips SM , Padgett LS , Leisenring WM , et al. Survivors of childhood cancer in the United States: prevalence and burden of morbidity. Cancer Epidemiol Biomarkers Prev. 2015;24(4):653‐663. 25834148 10.1158/1055-9965.EPI-14-1418 PMC4418452

[4] Hjorth L , Haupt R , Skinner R , et al. Survivorship after childhood cancer: PanCare: a European network to promote optimal long‐term care. Eur J Cancer. 2015;51(10):1203‐1211. 25958037 10.1016/j.ejca.2015.04.002 PMC5916870

[5] Childhood Cancer in Ontario: The 2020 POGO Surveillance Report. Pediatric Oncology Group of Ontario; 2020.

[6] Oeffinger KC , Mertens AC , Sklar CA , et al. Chronic health conditions in adult survivors of childhood cancer. N Engl J Med. 2006;355(15):1572‐1582. 17035650 10.1056/NEJMsa060185

[7] Hudson MM , Ness KK , Gurney JG , et al. Clinical ascertainment of health outcomes among adults treated for childhood cancer. JAMA. 2013;309(22):2371‐2381. 23757085 10.1001/jama.2013.6296 PMC3771083

[8] Frederiksen LE , Mader L , Feychting M , et al. Surviving childhood cancer: a systematic review of studies on risk and determinants of adverse socioeconomic outcomes. Int J Cancer. 2019;144(8):1796‐1823. 30098012 10.1002/ijc.31789

[9] Schulte F , Kunin‐Batson AS , Olson‐Bullis BA , et al. Social attainment in survivors of pediatric central nervous system tumors: a systematic review and meta‐analysis from the children's oncology group. J Cancer Surviv. 2019;13(6):921‐931. 31625086 10.1007/s11764-019-00808-3 PMC6900341

[10] Iyer NS , Balsamo LM , Bracken MB , Kadan‐Lottick NS . Chemotherapy‐only treatment effects on long‐term neurocognitive functioning in childhood ALL survivors: a review and meta‐analysis. Blood. 2015;126(3):346‐353. 26048910 10.1182/blood-2015-02-627414

[11] Macartney G , Harrison MB , VanDenKerkhof E , Stacey D , McCarthy P . Quality of life and symptoms in pediatric brain tumor survivors: a systematic review. J Pediatr Oncol Nurs. 2014;31(2):65‐77. 24608699 10.1177/1043454213520191

[12] Brier MJ , Schwartz LA , Kazak AE . Psychosocial, health‐promotion, and neurocognitive interventions for survivors of childhood cancer: a systematic review. Health Psychol. 2015;34(2):130‐148. 25133839 10.1037/hea0000119

[13] The POGO School and Work Transitions Program [SWTP Website]. https://www.pogo.ca/programs‐support/survivor‐care/transitions‐school‐work‐counselling/

[14] Boydell KM , Stasiulis E , Greenberg M , Greenberg C , Spiegler B . I'll show them: the social construction of (in)competence in survivors of childhood brain tumors. J Pediatr Oncol Nurs. 2008;25(3):164‐174. 18353751 10.1177/1043454208315547

[15] Stasiulis E , Boydell KM , Edelstein K , et al. The Transition to Meaningful Activity for Childhood Cancer Survivors: Understanding the Role of the POGO School and Work Transitions Program. The Pediatric Oncology Group of Ontario; 2020.

[16] Boydell K , Stasiulis E . An Academic Vocational Transition Program: Perspectives of Survivors of Childhood Cancer and their Families. The Pediatric Oncology Group of Ontario; 2003.

[17] Kiresuk TJ , Smith A , Cardillo JE , eds. Goal Attainment Scaling: Applications, Theory, and Measurement. Lawrence Erlbaum Associates, Inc; 1994.

[18] Richard NM , Bernstein LJ , Mason WP , et al. Cognitive rehabilitation for executive dysfunction in brain tumor patients: a pilot randomized controlled trial. J Neurooncol. 2019;142(3):565‐575. 30847839 10.1007/s11060-019-03130-1

[19] Harpster K , Sheehan A , Foster EA , Leffler E , Schwab SM , Angeli JM . The methodological application of goal attainment scaling in pediatric rehabilitation research: a systematic review. Disabil Rehabil. 2019;41(24):2855‐2864. 29954232 10.1080/09638288.2018.1474952

[20] van Seben R , Reichardt L , Smorenburg S , Buurman B . Goal‐setting instruments in geriatric rehabilitation: a systematic review. J Frailty Aging. 2017;6(1):37‐45. 28244557 10.14283/jfa.2016.103

[21] Stevens A , Beurskens A , Köke A , van der Weijden T . The use of patient‐specific measurement instruments in the process of goal‐setting: a systematic review of available instruments and their feasibility. Clin Rehabil. 2013;27(11):1005‐1019. 23881336 10.1177/0269215513490178

[22] Hurn J , Kneebone I , Cropley M . Goal setting as an outcome measure: a systematic review. Clin Rehabil. 2006;20(9):756‐772. 17005500 10.1177/0269215506070793

[23] Bovend'Eerdt TJ , Botell RE , Wade DT . Writing SMART rehabilitation goals and achieving goal attainment scaling: a practical guide. Clin Rehabil. 2009;23(4):352‐361. 19237435 10.1177/0269215508101741

[24] Kiresuk TJ , Sherman RE . Goal attainment scaling: a general method for evaluating comprehensive community mental health programs. Community Ment Health J. 1968;4(6):443‐453. 24185570 10.1007/BF01530764

[25] Tercyak KP , Donze JR , Prahlad S , Mosher RB , Shad AT . Identifying, recruiting, and enrolling adolescent survivors of childhood cancer into a randomized controlled trial of health promotion: preliminary experiences in the survivor health and resilience education (SHARE) program. J Pediatr Psychol. 2006;31(3):252‐261. 15758194 10.1093/jpepsy/jsj013

[26] Kilsdonk E , Wendel E , van Dulmen‐den BE , van Leeuwen FE , van den Berg MH , Jaspers MW . Participation rates of childhood cancer survivors to self‐administered questionnaires: a systematic review. Eur J Cancer Care (Engl). 2017;26(6):e12462. 10.1111/ecc.12462 26880318

[27] Ojha RP , Oancea SC , Ness KK , et al. Assessment of potential bias from non‐participation in a dynamic clinical cohort of long‐term childhood cancer survivors: results from the St. Jude lifetime cohort study. Pediatr Blood Cancer. 2013;60(5):856‐864. 23024097 10.1002/pbc.24348 PMC3548083

[28] Blaauwbroek R , Bouma MJ , Tuinier W , et al. The effect of exercise counselling with feedback from a pedometer on fatigue in adult survivors of childhood cancer: a pilot study. Support Care Cancer. 2009;17(8):1041‐1048. 19015892 10.1007/s00520-008-0533-y PMC2707951

[29] Rey‐Casserly C , Diver T . Late effects of pediatric brain tumors. Curr Opin Pediatr. 2019;31(6):789‐796. 31693589 10.1097/MOP.0000000000000837

[30] Mitby PA , Robison LL , Whitton JA , et al. Utilization of special education services and educational attainment among long‐term survivors of childhood cancer: a report from the childhood cancer survivor study. Cancer. 2003;97(4):1115‐1126. 12569614 10.1002/cncr.11117

[31] Lindahl M , Addington SV , Winther JF , Schmiegelow K , Andersen KK . Socioeconomic factors and ninth grade school performance in childhood leukemia and CNS tumor survivors. JNCI Cancer Spectr. 2018;2(1):pky003. 10.1093/jncics/pky003 PMC6649790 31360837

[32] Anderson VA , Godber T , Smibert E , Weiskop S , Ekert H . Cognitive and academic outcome following cranial irradiation and chemotherapy in children: a longitudinal study. Br J Cancer. 2000;82(2):255‐262. 10646874 10.1054/bjoc.1999.0912 PMC2363266

[33] Barrera M , Shaw AK , Speechley KN , Maunsell E , Pogany L . Educational and social late effects of childhood cancer and related clinical, personal, and familial characteristics. Cancer. 2005;104(8):1751‐1760. 16130127 10.1002/cncr.21390

[34] Boman KK , Bodegard G . Life after cancer in childhood: social adjustment and educational and vocational status of young‐adult survivors. J Pediatr Hematol Oncol. 2004;26(6):354‐362. 15167348 10.1097/00043426-200406000-00005

[35] Boman KK , Lindblad F , Hjern A . Long‐term outcomes of childhood cancer survivors in Sweden: a population‐based study of education, employment, and income. Cancer. 2010;116(5):1385‐1391. 20087961 10.1002/cncr.24840

[36] Dieluweit U , Debatin KM , Grabow D , et al. Educational and vocational achievement among long‐term survivors of adolescent cancer in Germany. Pediatr Blood Cancer. 2011;56(3):432‐438. 21072822 10.1002/pbc.22806

[37] Gilleland Marchak J , Devine KA , Hudson MM , et al. Systematic review of educational supports of pediatric cancer survivors: current approaches and future directions. J Clin Oncol. 2021;39(16):1813‐1823. 33886350 10.1200/JCO.20.02471

